# CX3CL1 Induces Vertebral Microvascular Barrier Dysfunction via the Src/P115-RhoGEF/ROCK Signaling Pathway

**DOI:** 10.3389/fncel.2020.00096

**Published:** 2020-04-24

**Authors:** Lei Yi, Yun Liang, Quanming Zhao, Houlei Wang, Jian Dong

**Affiliations:** ^1^Department of Burn and Plastic Surgery, School of Medicine, Ruijin Hospital, Shanghai Jiao Tong University, Shanghai, China; ^2^Department of Orthopedic Surgery, Zhongshan Hospital, Fudan University, Shanghai, China; ^3^Department of Orthopedic Surgery, The Second Affiliated Hospital of Nantong University, Nantong, China

**Keywords:** CX3CL1, endothelial cell, F-actin, ZO-1, Src, P115-RhoGEF, ROCK, spine

## Abstract

Trans-endothelial migration (TEM) of cancer cells is a critical step in metastasis. Micro-vascular barrier disruptions of distant organs play important roles in tumor cells TEM. The spine is a preferred site for multiple cancer cell metastases. Our previous study found that vertebral spongy bone was rich in CX3CL1 and that CX3CL1 can attract fractalkine receptor-expressing tumor cells to the spine. In the present study, we determined whether CX3CL1 was involved in vertebral micro-vascular barrier disruption and promoted tumor cell TEM after circulating tumor cells were arrested in the vertebral micro-vasculature. We examined the role of CX3CL1 in the barrier function of vertebral micro-vascular endothelial cells (VMECs) and explored the molecular mechanisms of CX3CL1-induced VMEC barrier disruption. Our results demonstrated that CX3CL1 led to F-actin formation and ZO-1 disruption in VMECs and induced the vertebral micro-vascular barrier disruption. Importantly, we found that the activation of the Src/P115-RhoGEF/ROCK signaling pathway plays an important role in CX3CL1-induced VMEC stress fiber formation, ZO-1 disruption and then vertebral micro-vascular barrier hyper-permeability. Inhibiting Src/P115-RhoGEF/ROCK signaling in VMECs effectively blocked CX3CL1-induced vertebral vascular endothelial dysfunction and subsequent tumor cell TEM. The results of this study and our previous study indicate that in addition to its chemotaxis, CX3CL1 plays a critical role in regulating vertebral micro-vascular barrier function and tumor cell TEM. CX3CL1 induced VMECs stress fiber formation, ZO-1 disruption and then vascular endothelial hyperpermeability via activation of the Src/P115-RhoGEF/ROCK signaling pathway. The inhibition of the Src/P115-RhoGEF/ROCK signaling pathway in VMECs effectively blocked tumor cells TEMs in vertebral spongy bone and maybe a potential therapeutic strategy for spine metastases in the future.

## Introduction

Metastasis is a fatal step in the progression of many malignancies. Vertebral cancellous bones, as a preferential district for bone metastases, are highly efficient at arresting circulating cancer cells from low-velocity bloodstream. Furthermore, CX3CL1, which is rich in vertebral microenvironments (Liu et al., 2017), chemo-attracts many tumor cells from blood circulation to the spine. CX3CL1 functions as an adhesion molecule for arresting circulating tumor cells in spine microvasculature (Ostuni et al., 2014; Zhou et al., 2016). However, the role of CX3CL1 in tumor cell trans-endothelial migration (TEM) in the spine remains unknown. Vertebral spongy bone is rich in micro-vessels. Vertebral micro-vascular endothelial cells (VMECs) line the surface of vascular sinusoids in the vertebral body to maintain vascular barrier integrity and spine homeostasis. After circulating cancer cells arrested in the microvasculature in the spine, the extravasation of circulating tumor cells into the vertebral spongy bone is a critical step for metastasis. In tumor cell TEM, microvascular endothelial barrier dysfunction and subsequent micro-vascular hyper-permeability promote metastasis formation. Interestingly, some studies have reported that CX3CL1 mediated vascular dysfunction in congestive heart failure (Hildemann et al., 2014). However, whether CX3CL1 induced VMECs injury and EC monolayer barrier disruption in the spine remains unclear. Vascular endothelial barrier function is primarily determined by cytoskeletal elements and cell-cell contact protein complex, such as tight junctions (TJs), in ECs (Dejana, 2004). The TJs component ZO-1 is a special intracellular junctional protein, which acted as adaptors in mediating the binding of adhesion proteins to actin (Dudek and Garcia, 2001). ZO-1 not only functioned as scaffold but also maintained the shape and polarity of ECs. Increased concentrations of actin filaments and ZO-1 disruption leads to ECs barrier disruption. Thus, in this study, we hypothesized that CX3CL1 induced the formation of stress fiber, the ZO-1 disruption in VMECs and then promoted VMECs barrier disruption, which further mediated vertebral micro-vascular hyper-permeability and increased tumor cell TEM in the spine.

Src family kinases (SFKs) are the largest family of non-receptor tyrosine kinases, which play an important role in proliferation, apoptosis, angiogenesis, and the regulation of vascular barrier function (Lehr et al., 2000). Src is one of the most widely studied members of the SFKs. Our previous studies found that CX3CL1 quickly activated Src signaling in several tumor cells (Liang et al., 2018; Liu et al., 2018). Interestingly, another study reported that Src regulated cytoskeleton remodeling in Schwann cell morphology and motility (Melfi et al., 2017). Moreover, Src is known to mediate microvascular leakage in response to lipopolysaccharide (LPS) stimulation. Inhibition of Src protein effectively blocked the increase in vascular permeability caused by C5a-activated neutrophils (Tinsley et al., 2002). The above data suggested that CX3CL1 might activate Src signaling in VMECs and regulate cellular cytoskeleton and vascular barrier function. However, the molecular mechanisms by which CX3CL1 activates the Scr signaling pathway and stress fiber formation in VMECs remain unclear.

RhoA/Rho-associated protein kinase (ROCK), acting as molecular switches to control many cellular functions (Yi et al., 2016a, b), is a critical regulator of the actomyosin contraction in ECs (Lee and Gotlieb, 2003; Birukova et al., 2004b) and positively regulates endothelial barrier dysfunction under sepsis (Lum and Malik, 1996; Bogatcheva et al., 2011). P115-RhoGEF and GEF-H1 appear to be involved in the activation of RhoA/ROCK (Zheng, 2001; Ying et al., 2004; Garcia et al., 2009). Recent studies showed that tensions on junctional adhesion molecule A (JAM-A) activated RhoA/ROCK via Src/p115-RhoGEF signaling (Scott et al., 2016). Given the close connection among Src, P115-RhoGEF, ROCK and endothelial barrier function, in this study, we aimed to determine whether CX3CL1 induced VMEC stress fiber formation, ZO-1 disruption and then micro-vascular barrier disruption via activation of the Src/p115-RhoGEF/ROCK signaling pathway.

**FIGURE 1:**
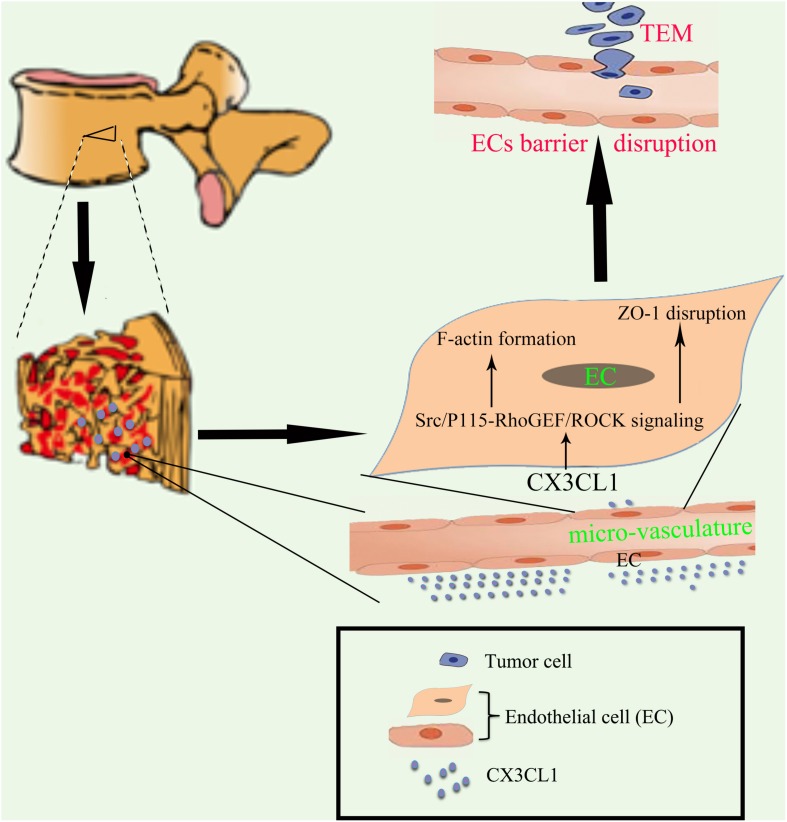
Proposed schematic representation of how CX3CL1 induces microvascular barrier disruption in the spine. The vertebral spongy bone is rich in CX3CL1. CX3CL1 induces activation of the Src/P115-RhoGEF/ROCK signaling pathway in VMECs, which further induces the F-actin formation and ZO-1 disruption in ECs and subsequent microvascular barrier disruption, resulting in exacerbation of tumor cells TEM in the spine.

In the present study, we found that CX3CL1 induced VMEC barrier disruption in a time- and dose-dependent manner. We further demonstrated that the CX3CL1-induced VMEC barrier dysfunction was due to the activation of Src/p115-RhoGEF/ROCK signaling and subsequent stress fiber formation and ZO-1 disruption in VMECs. Importantly, the inhibition of Src/p115 RhoGEF/ROCK signaling in VMECs not only blocked CX3CL1-induced VMEC monolayer hyper-permeability but also inhibited tumor cell TEM (Figure 1). Our findings revealed the molecular mechanisms of CX3CL1-induced VMEC barrier disruption and provided new insight into the role of CX3CL1 on tumor cells TEM via its regulatory effects on vertebral micro-vasculature, which may provide new therapeutic directions for circulating tumor cell TEM in the spine.

## Materials and Methods

### Cell Culture and Reagents

Vertebral micro-vascular endothelial cells (VMECs) were obtained from the spine of C57BL/6 mice (8–10 weeks old, 20–25 g) as previously described (Yu et al., 2015) and were maintained in ScienCell Endothelial Cell Medium (Cat. No. 1001; ECM consisted of 500 ml of basal medium, 25 ml of fetal bovine serum, 5 ml of endothelial cell growth supplement and 5 ml of penicillin/streptomycin solution) in a humidified 37°C, 5% CO_2_ incubator. Two cancer cell types (A549-Luciferase-GFP and 786-O-Luciferase-mCherry) were obtained from the Chinese Academy of Sciences Cell Bank (Shanghai, China). The cancer cells were cultured in RPMI-1640 media (Cat. No. 61870044; Gibco; Thermo Fisher Scientific, Inc.), and the complete media contained 10% fetal bovine serum (Cat. No. 10099141; Gibco; Thermo Fisher Scientific, Inc.) and 1%penicillin/streptomycin (Cat. No. B557; Invitrogen; Thermo Fisher Scientific, Inc.).

CX3CL1 was purchased from PeproTech (New Jersey, United States), and Bosutinib (Src inhibitor) was purchased from Selleck (Houston, TX, United States). Anti-Src rabbit monoclonal antibody (mAb), anti-phospho-Src (Tyr416) rabbit mAb, anti-phospho- MLC (Myosin Light Chain) (Thr18/Ser19) rabbit mAb, anti-P115 RhoGEF rabbit mAb, anti-GEF-H1 rabbit mAb, anti-ZO-1 rabbit mAb, and anti-rabbit immunoglobulin-G-HRP-linked antibody were bought from Cell Signaling Technologies (Danvers, MA, United States). Phalloidin, Alexa Fluor Plus 555-conjugated donkey anti-rabbit secondary antibody and Prolong Gold Antifade Reagent with DAPI were obtained from Invitrogen Life Science.

### ECIS Measurements

The ECs barrier function was monitored by ECIS as previously described (Yi et al., 2017). Endothelial monolayer resistance was monitored with the ECIS (Electric Cell-substrate Impedance Sensing) method using the ECIS 1,600 instrument and 8W10E electrode arrays. All equipment was purchased from ibidi GmbH in cooperation with Applied Bio-Physics (Martinsried, Germany). ECs were grown on 8-well electrode arrays. CX3CL1 (0.01, 0.1, and 1 μg/ml) was added when cells were confluent, showing a stable plateau of resistance value on a connected monitor. The changes in transendothelial electrical resistance were monitored continuously by the ECIS.

### Western Blot Assay

VMECs were seeded in 6-well plates and then stimulated in the culture medium with the following stimulation of CX3CL1 (1 μg/ml). Treated ECs were harvested and lysed for 30 min in pre-cold PIPA + PMSF (100:1). After centrifugation, the supernatants were collected as to total cellular protein extracts, and the protein concentration was measured using the Bradford method (Pierce BCA Protein Assay Kit, No. 23225; Thermo Fisher Scientific, Rockford, IL, United States) according to the manufacturer protocols. Whole cellular protein extracts were separated using 6–12% sodium dodecyl sulfate-polyacrylamide gel electrophoresis, and the proteins were electro-transferred to an Immobilon-P Transfer Membrane (Millipore, Billerica, MA, United States) by a wet transferor. The membranes were incubated with primary antibodies (e.g., against Src) and subsequently incubated with the appropriate HRP-linked secondary antibodies. Signal intensities were compensated by GAPDH or ACTIN as internal controls. Finally, the bands of specific proteins on the membranes were developed with Western Blotting Luminal Reagent (Millipore, Billerica, MA, United States) according to the manufacturer’s instructions.

### Transfection With P115-RhoGEF siRNA

Stealth RNA interference duplexes against mouse P115-RhoGEF were designated and synthesized by Genepharma Technologies (Shanghai, China). P115-RhoGEF small interfering RNA (siRNA) molecules were individually transfected into VMECs at a 50 nmol/L concentration using Lipofectamine 3000 (Carlsbad, CA, United States) according to the manufacturer’s instructions. The ability of the RNA interference molecules to knockdown target protein expression was analyzed at 48 h after transfection by Western blot analysis. Transfections of FITC-labeled nonspecific siRNA showed up to 85% transfection efficiency in VMECs.

### Confocal Immunofluorescent Analysis

ECs were grown to form EC monolayer on glass slide. After the indicated treatments, ECs were rinsed in PBS, fixed in 4% formaldehyde, and incubated with primary antibody against ZO-1 (1:400) overnight at 4°C. ECs were then washed, incubated with Alexa Fluor Plus 555-conjugated donkey anti-rabbit secondary antibody (1:1000) and mounted in Prolong Gold Antifade Reagent with DAPI. Quantification of CX3CL1-induced tight junction protein disruption was performed as described elsewhere (Birukova et al., 2016).

For actin staining, VMECs were washed twice with pre-warmed PBS (pH 7.4), fixed in 4% paraformaldehyde for 20 min, and blocked for 30 min with PBS containing 1% bovine serum albumin, followed by 20 min of incubation with FITC-labeled phalloidin (5:200). The fluorescence images of F-actin under glass coverslips were captured on a Nikon Eclipse E400 were captured by a microscope (Nikon, Tokyo, Japan). Nuclei of VMECs were counterstained by DAPI. Quantitative analysis of stress fiber formation was performed as described (Birukova et al., 2004a).

### Assessment of Albumin Leak Across the ECs Monolayer

EC barrier leak was assessed by EB-albumin as previously described (Shelton et al., 2006). VMECs were grown to confluence on gelatine-coated Transwell inserts (0.4 μm pore size, Corning, Inc., Corning, NY, United States) in 24-well plates and then stimulated with CX3CL1. Subsequently, 0.75 ml of a 4% solution of albumin was added to the lower compartment of the well and 0.5 ml of EB-albumin solution was added to the upper compartment. The amount of EB-albumin that leaked across the VMEC monolayer into the lower compartment of the Transwell inserts was quantified by measuring the absorbance of the medium in the lower compartment at 595 nm. Trans-EC EB-albumin flux is expressed as the percentage of total EB-albumin added to the upper compartment.

### Trans-Endothelial Migration (TEM) of Tumor Cell

A total 10^5^ ECs were plated in the upper chamber of Transwell insert and grown in EC medium to form EC monolayer. After stimulating with or without CX3CL1, the fluorescently labeled tumor cells were plated on top of the endothelial monolayer. The cells were allowed to migrate for the indicated time in cell incubator. The transwells were then fixed in 4% paraformaldehyde, the cells on the apical side of each insert were scraped off, and the transwell membrane mounted onto glass slides. The tumor cells migrating to the basal side of the membrane were visualized with immunofluorescent microscope. Quantitative analysis of migration rate of tumor cell was performed as described (Tichet et al., 2015).

### Statistical Analysis

All results are expressed as the means ± SEM of at least 3 independent experiments. Data are expressed as means and standard errors. Student’s *t*-tests and ANOVAs were used as appropriate. Significance was accepted at *P* < 0.05.

## Results

### CX3CL1 Induces VMEC Barrier Disruption in a Dose- and Time-Dependent Manner

**FIGURE 2:**
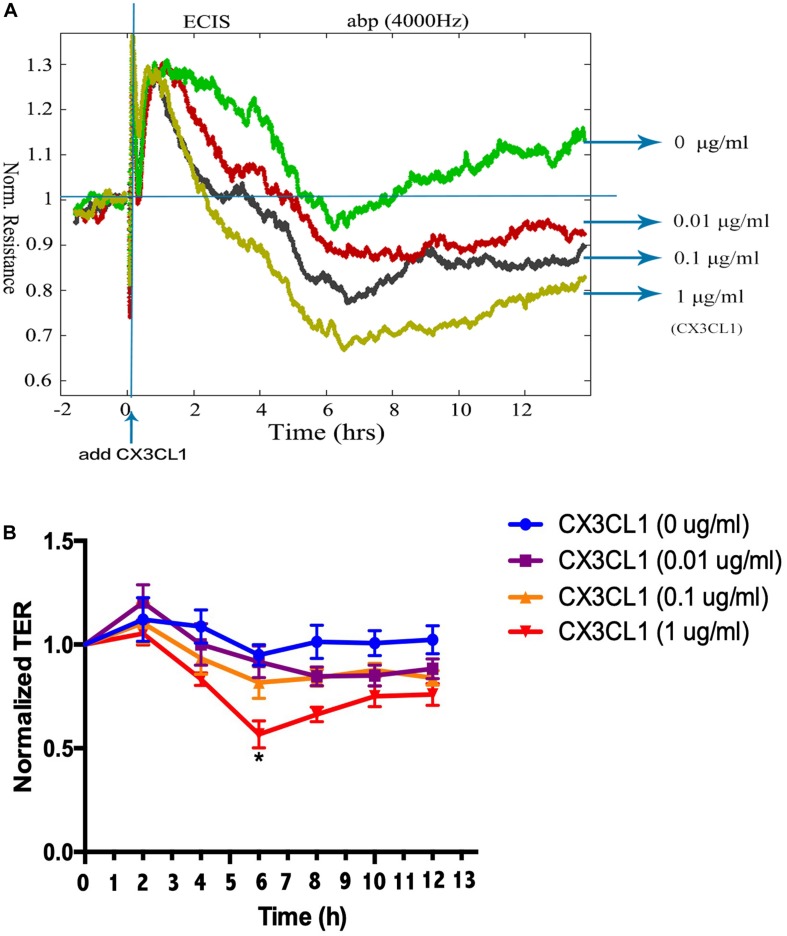
CX3CL1 induces VMEC monolayer barrier disfunction. The VMECs were plated on the gold microelectrodes. **(A)** When VMECs formed a monolayer, and the TER values reached stable, different concentrations of CX3CL1 (0, 0.01, 0.1, 1 μg/ml) was added. The VMEC monolayer permeability was determined by real-time TER measurement. **(B)** The results of normalized TER were represented as curve graph. *P < 0.05 vs. the negative control.

To determine the underlying roles of CX3CL1 on the integrity of VMEC monolayers, ECs were stimulated with different concentrations of CX3CL1 (0, 0.01, 0.1, and 1 μg/ml), and the trans-endothelial electrical resistance (TER) was continuously monitored. The measurements revealed that CX3CL1 induced VMEC barrier disruption, TER decreased in a dose- and time-dependent manner and peak EC barrier disruption appeared at 6 h after stimulation with 1 μg/ml CX3CL1 (Figures 2A,B).

### CX3CL1 Induces VMEC Barrier Disruption via EC Cytoskeletal Rearrangement

**FIGURE 3:**
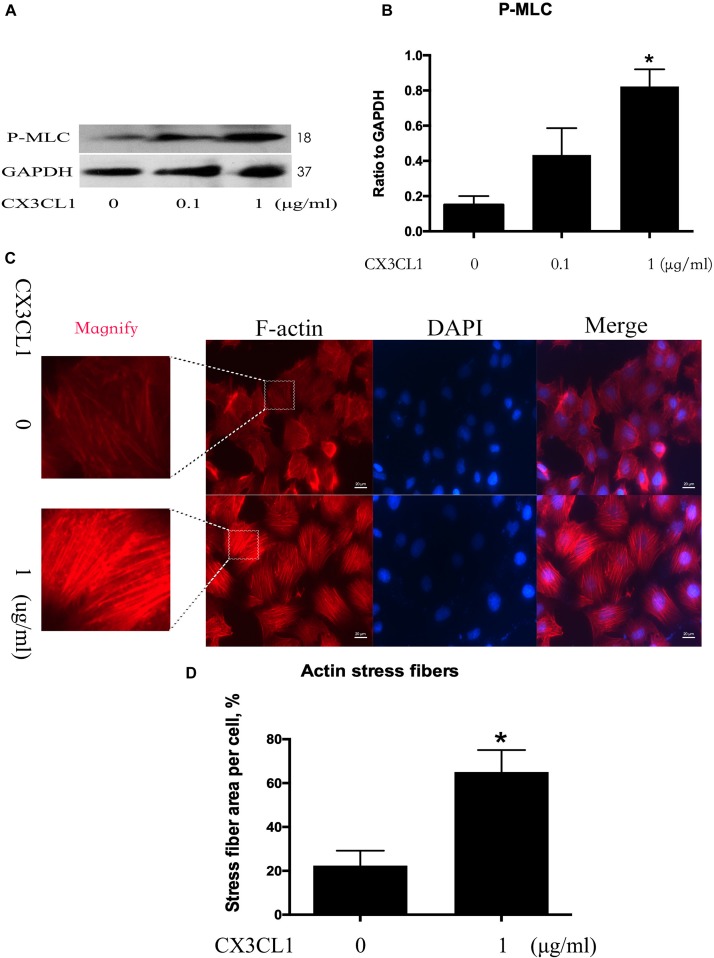
CX3CL1 induces F-actin and stress fiber formation in VMECs. **(A)** The expression of P-MLC was examined at the indicated time point after CX3CL1 (1 μg/ml) stimulation in VMECs. **(B)** The results were represented as a histogram according to band intensities. **(C)** Immunofluorescence analysis of F-actin formation in the VMECs. **(D)** Data are expressed as a ratio of the intracellular area occupied by stress fibers to the whole cell area. *P < 0.05 vs. the negative control.

Cytoskeletal rearrangement and stress fiber formation induced EC contraction and subsequent EC barrier disruption. MLC (Myosin Light Chain) phosphorylation plays an important role in myosin filament assembly and stress fiber formation (Kawano et al., 1999; McKenzie and Ridley, 2007). We incubated VMECs with various concentrations of CX3CL1 (0, 0.1, and 1 μg/ml) for 6 h and found that the P-MLC expression exhibited an increasing trend with increasing concentrations of CX3CL1 (Figures 3A,B). VMECs cytoskeletal remodeling after stimulation with CX3CL1 was analyzed by immunofluorescence staining of F-actin. CX3CL1 (1 μg/ml) incubation with for 6 h induced stress fiber formation and EC contraction (Figures 3C,D). These data were consistent with those presented in Figure 2 and demonstrated that CX3CL1 induced VMECs barrier dysfunction via F-actin formation.

### CX3CL1 Induces VMEC Barrier Disruption via ZO-1 Disruption

**FIGURE 4:**
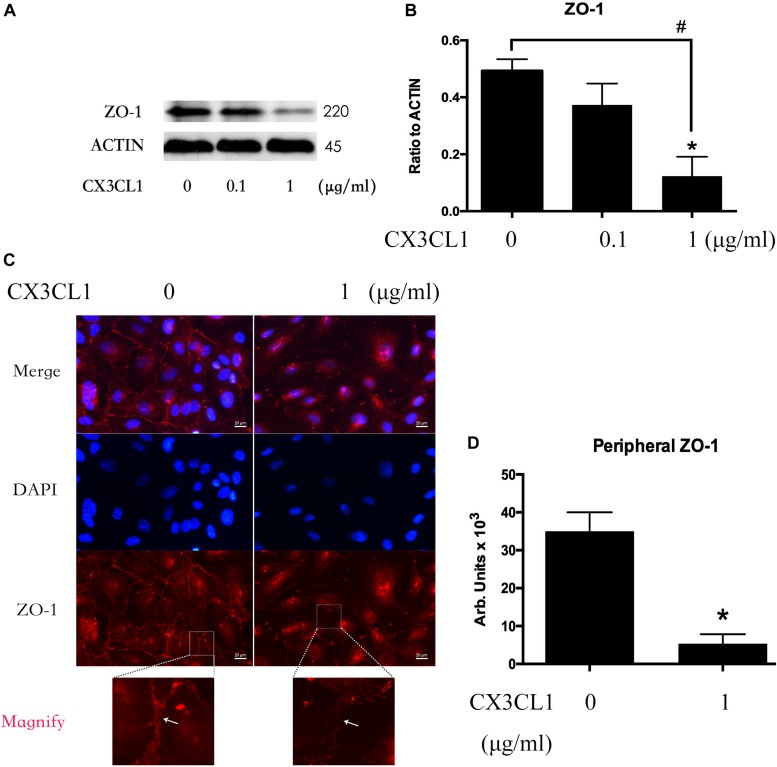
CX3CL1 induces ZO-1 disruption in VMECs. **(A)** The expression of ZO-1 was examined at the indicated time point after CX3CL1 (1 μg/ml) stimulation in VMECs. **(B)** The results were represented as a histogram according to band intensities. **(C)** Immunofluorescence analysis of ZO-1 in the VMECs. **(D)** The results were represented as a histogram according to measurement of the integrated fluorescence density in the peripheral area. *P < 0.05 vs. the negative control.

We incubated VMECs with various concentrations of CX3CL1 (0, 0.1, and 1 μg/ml) for 6 h and found that the ZO-1 expression exhibited an decreasing trend with increasing concentrations of CX3CL1 (Figures 4A,B). The cell-cell contact protein complex disruption in VMECs after stimulation with CX3CL1 was analyzed by immunofluorescence staining of ZO-1. CX3CL1 (1 μg/ml) incubation with for 6 h induced ZO-1 disruption and EC monolayer barrier dysfunction (Figures 4C,D). These data were consistent with those presented in Figure 2 and demonstrated that CX3CL1 also induced VMECs barrier dysfunction via ZO-1 disruption.

### CX3CL1 Induces Src Signaling Activation in a Dose- and Time-Dependent Manners in VMECs

**FIGURE 5:**
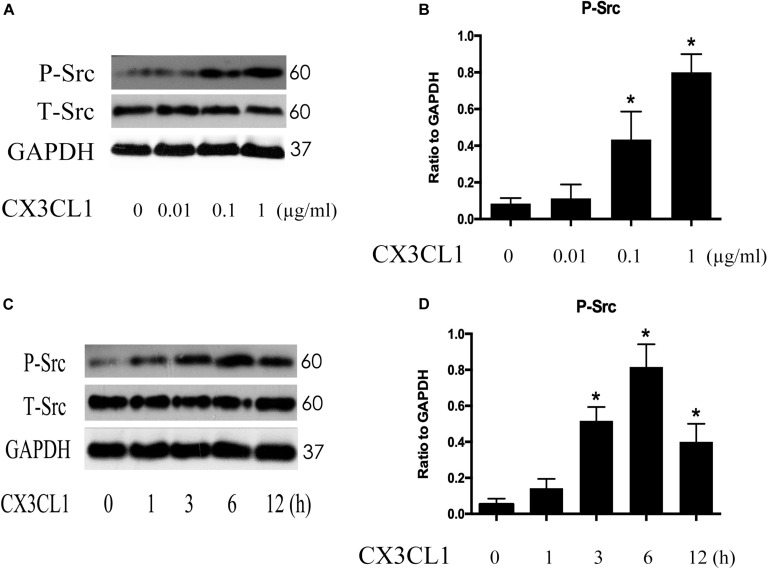
CX3CL1 induces Src activation in VMECs. **(A)** P-Src was detected after stimulation with different concentrations of CX3CL1 (0, 0.01, 0.1, 1 μg/ml) for 6 h, and the peak appeared at a concentration of 1 μg/ml. **(B)** The results were represented as a histogram according to band intensities. **(C)** VMECs were stimulated with CX3CL1 (1 μg/ml) at the different indicated times, and the peak appeared at 6 h. **(D)** The results were represented as a histogram according to band intensities. *P < 0.05 vs. the negative control.

Src signaling plays an important role in cell skeleton rearrangement and migration. To determine whether Src signaling is involved in CX3CL1-induced VMECs F-actin formation, we analyzed Src protein activity changes after stimulating VMECs with CX3CL1 by assessing the expression of Src phosphorylated on tyrosine 416 (Hamaguchi et al., 1995). VMECs were stimulated with various concentrations of CX3CL1 (0, 0.01, 0.1, and 1 μg/ml) for 6 h; then, the expression of total Src protein and P-Src protein was determined by Western blot. After incubating VMECs with CX3CL1, P-Src expression increased in a dose-dependent manner (Figures 5A,B). Subsequently, VMECs were exposed to CX3CL1 (1 μg/ml) for different times. Immunoblot assays revealed that LPS increased P-Src expression in a time-dependent manner (Figures 5C,D). These data indicated that CX3CL1 induced Src activation in a dose- and time-dependent manner in VMECs and that the peak of Src signaling activation appeared at 6 h after CX3CL1 (1 μg/ml) stimulation.

### Src Signaling Activation Is Involved in CX3CL1-Induced VMECs Stress Fiber Formation and ECs Barrier Disruption

**FIGURE 6:**
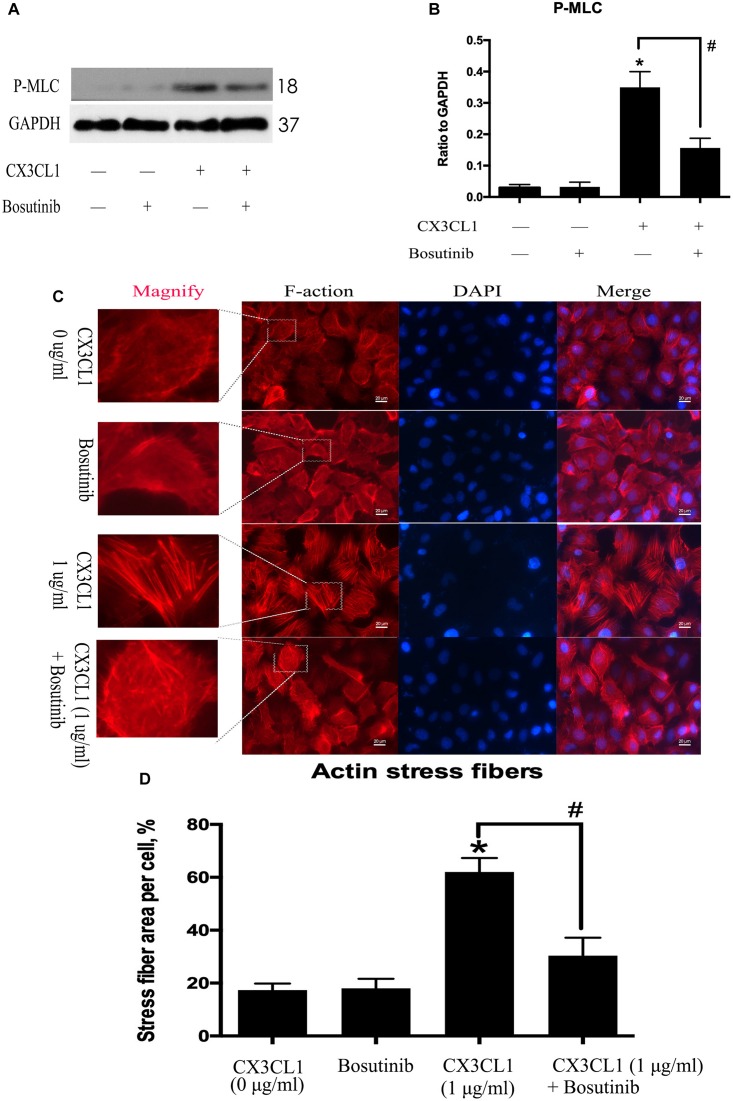
Effect of Src signaling on the CX3CL1-induced F-actin formation. **(A)** VMECs were treated with Bosutinib (2 μM) for 1h before CX3CL1 stimulation, and the expression of P-MLC was determined by Western blot. **(B)** The results were represented as a histogram according to band intensities. **(C)** VMECs were incubated with Bosutinib (2 μM) and/or CX3CL1 (1 μg/ml) before fixing and staining with the phalloidin. F-actin (red) and DAPI (blue) were examined by immunofluorescence microscopy. **(D)** Data are expressed as a ratio of the intracellular area occupied by stress fibers to the whole cell area. *P < 0.05 vs. the negative control. ^#^*P* < 0.05 vs. the corresponding CX3CL1.

CX3CL1-induced Src activation exhibited a similar trend to CX3CL1-induced VMEC F-actin formation and EC barrier disruption. We further used Bosutinib (a special inhibitor of Src) to analyze the effect of Src on CX3CL1-induced stress fiber formation in VMECs. We found that inhibiting Src activation effectively blocked CX3CL1-induced P-MLC expression (Figures 6A,B). We also found that pretreating VMECs with Bosutinib significantly inhibited CX3CL1-induced VMEC cytoskeleton rearrangement and cell contraction at the indicated times (Figures 6C,D). These results indicated that Src signaling activation plays an important role in CX3CL1-mediated stress fiber formation in VMECs and EC barrier disruption.

### Src Signaling Activation Is Involved in CX3CL1-Induced ZO-1 Disruption in VMEC and EC Monolayer Barrier Disfunction

**FIGURE 7:**
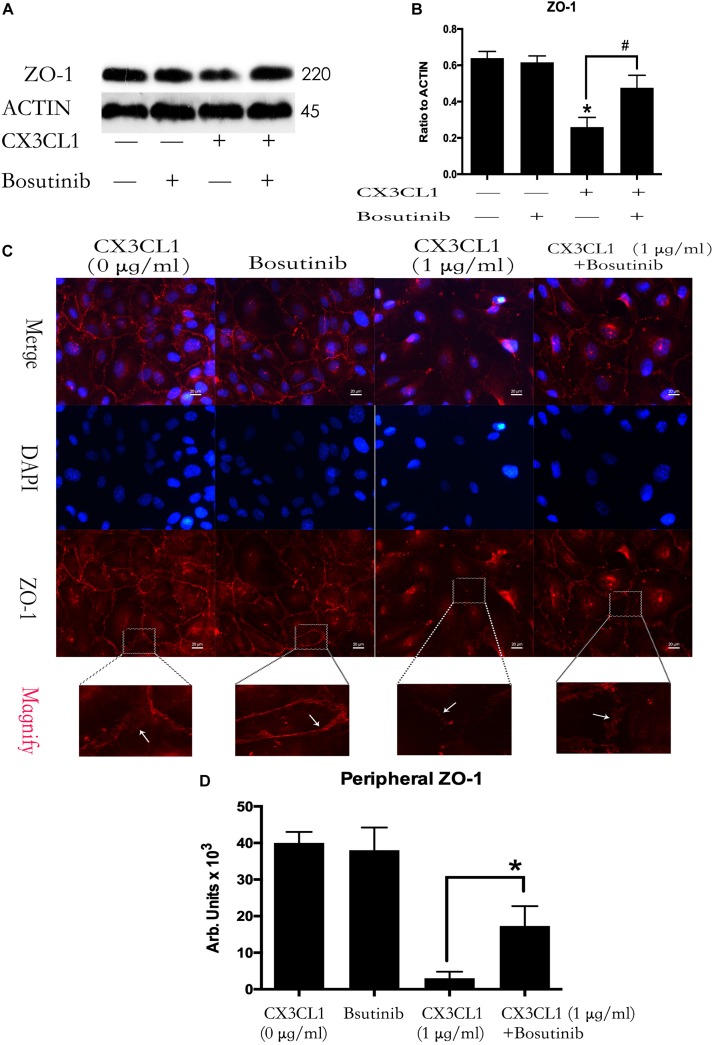
Effect of Src signaling on the CX3CL1-induced ZO-1 disruption. **(A)** VMECs were treated with Bosutinib (2 μM) for 1h before CX3CL1 stimulation, and the expression of ZO-1 was determined by Western blot. **(B)** The results were represented as a histogram according to band intensities. **(C)** VMECs were incubated with Bosutinib (2 μM) and/or CX3CL1 (1 μg/ml) before fixing and staining with the ZO-1. ZO-1 (red) and DAPI (blue) were examined by immunofluorescence microscopy. **(D)** The results were represented as a histogram according to measurement of the integrated fluorescence density in the peripheral area. *P < 0.05 vs. the negative control. ^#^*P* < 0.05 vs. the corresponding CX3CL1.

We used Bosutinib (a special inhibitor of Src) to analyze the effect of Src on CX3CL1-induced ZO-1 expression in VMEC. We found that inhibiting Src activation effectively blocked CX3CL1-induced decrease of ZO-1 expression (Figures 7A,B). We also found that pretreating VMECs with Bosutinib significantly inhibited CX3CL1-induced VMEC ZO-1 disruption and EC monolayer barrier disfunction at the indicated times (Figures 7C,D). These results indicated that Src signaling activation plays an important role in CX3CL1-mediated ZO-1 disruption in VMECs and EC barrier disruption.

### CX3CL1 Induces Both P115-RhoGEF/ROCK Signaling and GEF-H1/ROCK Signaling Activation in VMECs

**FIGURE 8:**
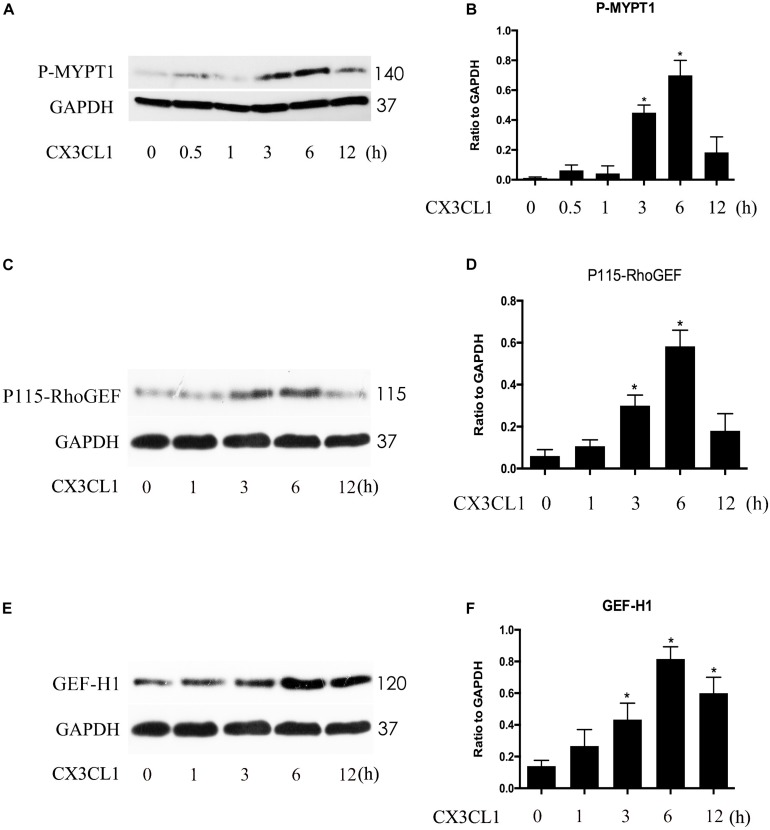
CX3CL1 induced activation of P115-RhoGEF/ROCK and GEF-H1/ROCK signaling in a time-dependent manner in VMECs. **(A)** The expression of P-MYPT at indicated time points after stimulation with CX3CL1 (1 μg/ml) in VMECs. **(B)** The results were represented as a histogram according to band intensities. **(C)** The expression of P115-RhoGEF at indicated time points after stimulation with CX3CL1 (1 μg/ml) in VMECs. **(D)** The results were represented as a histogram according to band intensities. **(E)** The expression of GEF-H1 at indicated time points after stimulation with CX3CL1 (1 μg/ml) in VMECs. **(F)** The results were represented as a histogram according to band intensities. *P < 0.05 vs. the negative control.

P115-RhoGEF and GEF-H1 play an important role in vascular endothelial dysfunction and ECs hyper-permeability (Birukova et al., 2007; Zhou et al., 2013). ROCK kinase is downstream of GEF proteins and acts as a key regulator of the initiation of ECs cytoskeleton contraction (Zhou et al., 2013). To determine whether P115-RhoGEF/ROCK or GEF-H1/ROCK signaling is involved in CX3CL1-induced VMECs stress fiber formation and EC contraction, we measured ROCK activity by assessing the expression of P-MYPT1 (Mali et al., 2011) and tested the expression of P115-RhoGEF and GEF-H1 via Western blot. We found that CX3CL1 (1 μg/ml) mediated the rapid activation of ROCK in a time-dependent manner in VMECs, peaking at 6 h (Figures 8A,B). We also found that CX3CL1 increased the expression of P115-RhoGEF (Figures 8C,D) and GEF-H1 (Figures 8E,F) in a time-dependent manner in VMECs, which coincided with the trend of Src signaling activation in CX3CL1-stimulated VMECs in Figure 4.

### CX3CL1 Induces Src/P115-RhoGEF/ROCK Signaling Activation in VMECs

**FIGURE 9:**
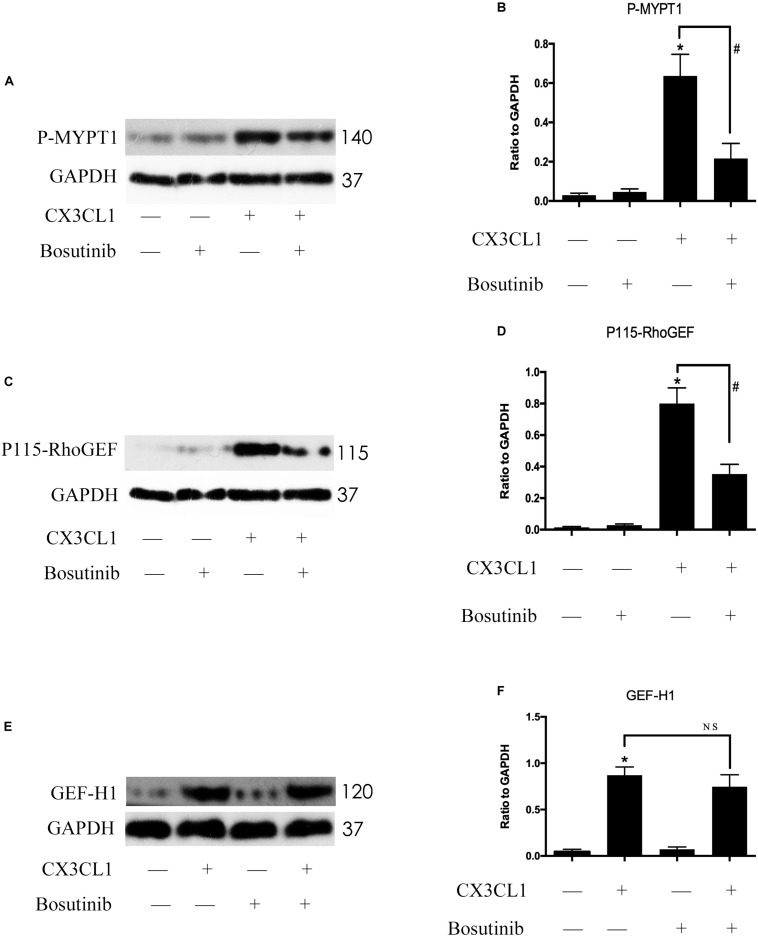
Src signaling does not block CX3CL1-induced GEF-H1 expression but inhibits CX3CL1-induced P115-RhoGEF signaling activation. **(A)** VMECs were incubated with Bosutinib (2 μM) for 1 h before CX3CL1 (1 μg/ml) stimulation for 6 h and the expression of P-MYPT1 was determined by Western blot. **(B)** The results were represented as a histogram according to band intensities. **(C)** VMECs were incubated with Bosutinib for 1 h before CX3CL1 stimulation for 3 h, and the expression of P115-RhoGEF was determined by Western blot. **(D)** The results were represented as a histogram according to band intensities. **(E)** VMECs were incubated with Bosutinib for 1h before CX3CL1 stimulation for 6h, and the expression of GEF-H1 was determined by Western blot. **(F)** The results were represented as a histogram according to band intensities. *P < 0.05 vs. the negative control. ^#^*P* < 0.05 vs. the corresponding CX3CL1. NS, no significance.

Previous studies reported that Src signaling could regulate the expression and activity of GEF proteins (Scott et al., 2016; Azoitei et al., 2019). To exploit the relationship between Src signaling pathway members, we examined the roles of P115-RhoGEF/ROCK and GEF-H1/ROCK signaling in CX3CL1-stimulated VMECs. We pretreated VMECs with Bosutinib for 1 h before incubating with CX3CL1 for 6 h to examine the changes to P115-RhoGEF/ROCK and GEF-H1/ROCK signaling. We found that inhibiting Src signaling not only blocked the CX3CL1-induced ROCK activation (Figures 9A,B) but also inhibited CX3CL1-induced P115-RhoGEF expression in VMECs (Figures 9C,D). However, pretreating VMECs with special Src inhibitor did not inhibit CX3CL1-induced GEF-H1 expression (Figures 9E,F). These data suggested that the Src/P115-RhoGEF/ROCK signaling was activated in VMECs after stimulating the cells with CX3CL1.

### P115-RhoGEF/ROCK Signaling Pathway Is Involved in CX3CL1-Induced VMECs Stress Fiber Formation

**FIGURE 10:**
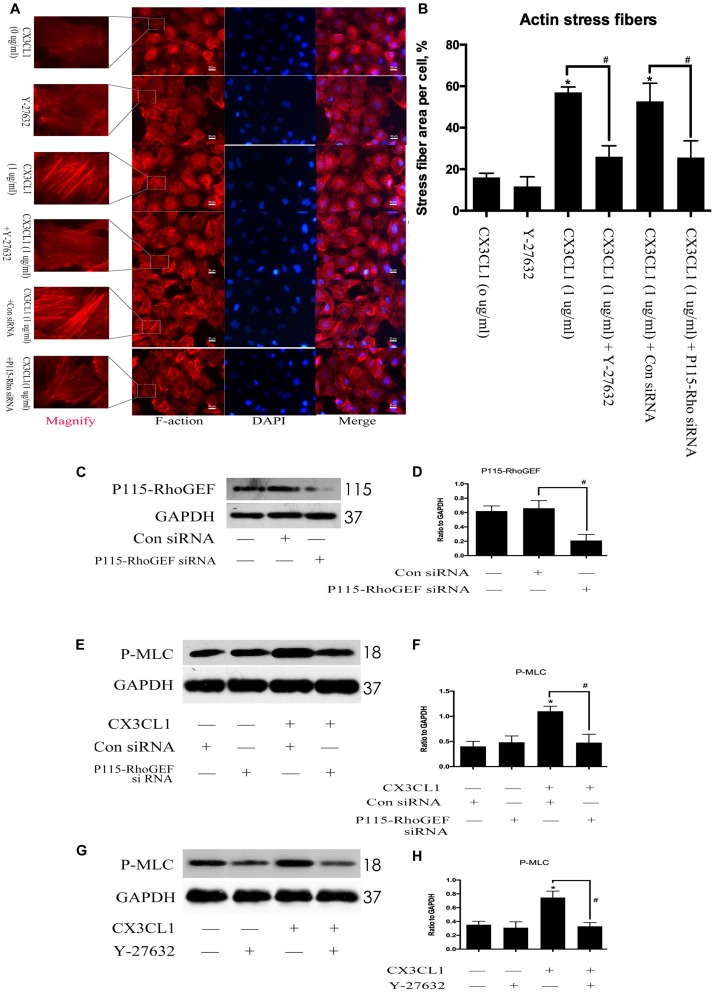
Effect of P115-RhoGEF/ROCK signaling on CX3CL1-induced F-actin formation. **(A)** VMECs were incubated with Y-27632 (10 μM), P115-RhoGEF, or CX3CL1 (1 μg/ml) before fixing and staining with the phalloidin. F-actin (red) and DAPI (blue) were examined by immunofluorescence microscopy. **(B)** Data are expressed as a ratio of the intracellular area occupied by stress fibers to the whole cell area. **(C)** The P115-RhoGEF knockdown effect was analyzed in VMECs by Western blot. **(D)** The results were represented as a histogram according to band intensities. #*P* < 0.05 vs. the control siRNA group. **(E)** VMECs were transferred with P115-RhoGEF siRNA or control siRNA for 6 h and then were stimulated with CX3CL1 (1 μg/ml) for 6 h. The expression of P-MLC was assessed by Western blot. **(F)** The results were represented as a histogram according to band intensities. **(G)** VMECs were treated with Y-27632 (10 μM) for 1 h before CX3CL1 stimulation, and the expression of P-MLC was determined by Western blot. **(H)** The results were represented as a histogram according to band intensities. *P < 0.05 vs. the negative control. ^#^*P* < 0.05 vs. the corresponding CX3CL1.

To determine whether P115-RhoGEF/ROCK signaling is involved in the regulation of CX3CL1-induced VMECs F-actin formation and ECs barrier function, we first incubated VMECs with Y-27632 for 1 h and then added CX3CL1 for another 6 h. We found that Y-27632 decreased CX3CL1-induced stress fiber formation in VMECs (Figures 10A,B). In addition, the VMECs were transfected with P115-RhoGEF siRNA or control siRNA for 6 h and then treated with medium or CX3CL1 for 6 h. The P115-RhoGEF knockdown was confirmed by Western blot assay (Figures 10C,D). In the present study, we found that P115-RhoGEF silencing effectively decreased CX3CL1-induced stress fiber formation in VMECs (Figures 10A,B). Furthermore, the effects of P115-RhoGEF/ROCK on CX3CL1-induced F-actin formation were confirmed by immunoblot assay. Compared with the control group, P115-RhoGEF silencing significantly inhibited the expression of P-MLC in CX3CL1-stimulated VMECs (Figures 10E,F). Inhibiting the activity of ROCK also effectively blocked CX3CL1-induced P-MLC expression in VMECs (Figures 10G,H). Taken together, these data combined with the results shown in Figure 6 illustrated that CX3CL1-induced VMECs stress fiber formation was dependent on the activation of the Src/P115-RhoGEF/ROCK signaling pathway.

### P115-RhoGEF/ROCK Signaling Pathway Is Involved in CX3CL1-Induced ZO-1 Disruption in VMEC

**FIGURE 11:**
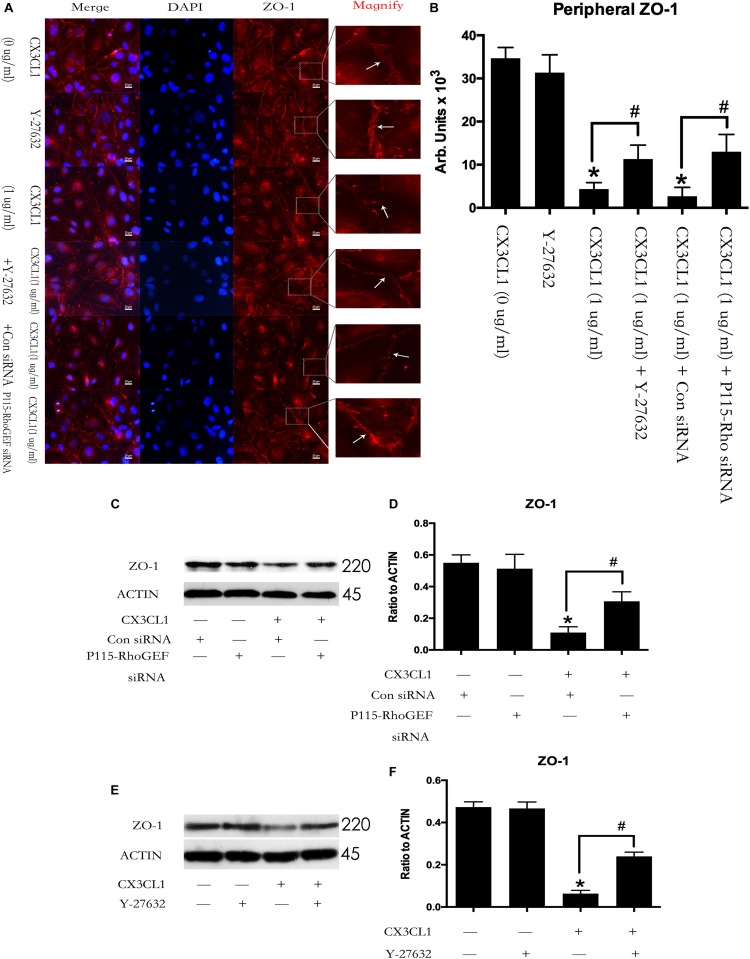
Effect of P115-RhoGEF/ROCK signaling on CX3CL1-induced ZO-1 disruption. **(A)** VMECs were incubated with Y-27632 (10 μM), P115-RhoGEF or CX3CL1 (1 μg/ml) before fixing and staining with the phalloidin. ZO-1 (red) and DAPI (blue) were examined by immunofluorescence microscopy. **(B)** The results were represented as a histogram according to measurement of the integrated fluorescence density in the peripheral area. **(C)** VMECs were transferred with P115-RhoGEF siRNA or control siRNA for 6 h and then were stimulated with CX3CL1 (1 μg/ml) for 6 h. The expression of ZO-1 was assessed by Western blot. **(D)** The results were represented as a histogram according to band intensities. **(E)** VMECs were treated with Y-27632 (10 μM) for 1h before CX3CL1 stimulation, and the expression of ZO-1 was determined by Western blot. **(F)** The results were represented as a histogram according to band intensities. *P < 0.05 vs. the negative control. ^#^*P* < 0.05 vs. the corresponding CX3CL1.

We first incubated VMECs with Y-27632 for 1 h and then added CX3CL1 for another 6 h. We found that Y-27632 reversed CX3CL1-induced ZO-1 disruption in VMECs (Figures 11A,B). In addition, the VMECs were transfected with P115-RhoGEF siRNA or control siRNA for 6 h and then treated with medium or CX3CL1 for 6 h. In the present study, we found that P115-RhoGEF silencing effectively blocked CX3CL1-induced ZO-1 disruption in VMECs (Figures 11A,B). Furthermore, the effects of P115-RhoGEF/ROCK on CX3CL1-induced F-actin formation were confirmed by immunoblot assay. Compared with the control group, P115-RhoGEF silencing significantly reversed the decrease of ZO-1 expression in CX3CL1-stimulated VMECs (Figures 11C,D). Inhibiting the activity of ROCK also effectively blocked CX3CL1-induced ZO-1 decrease in VMECs (Figures 11E,F).

### CX3CL1 Induces VMECs Barrier Disruption via Src/P115-RhoGEF/ROCK Signaling Activation

**FIGURE 12:**
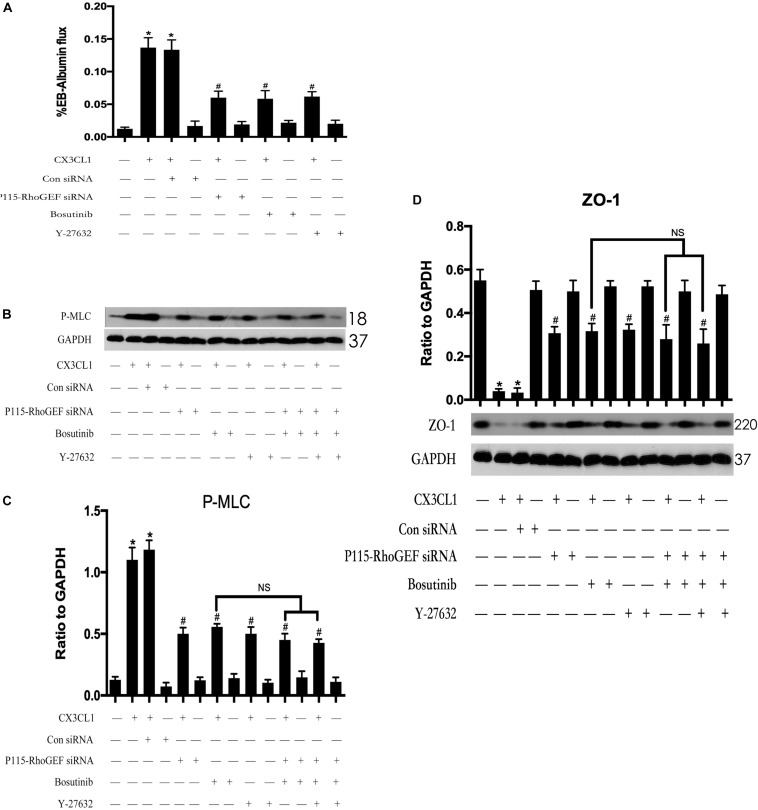
Effect of Src/P115-RhoGEF/ROCK signaling pathway on CX3CL1-induced VMECs barrier disruption. **(A)** After transfection with P115-RhoGEF siRNA for 48 h, VMECs were treated with CX3CL1 (1 μg/ml) for 6 h, and the ECs monolayer permeability was determined using EB-albumin in the Transwell and measuring the optical density at 595 nm in the lower chamber. Similarly, VMECs were pre-treated with Y-27632 (10 μM) or Bosutinib (2 μM) for 1h and then stimulated with CX3CL1 (1 μg/ml) for 6 h, and the ECs monolayer permeability was determined using EB-albumin in the Transwell chamber. **(B)** After transfection with P115-RhoGEF siRNA for 48 h, VMECs were treated with Y-27632 (10 μM) and/or Bosutinib (2 μM) for another 1h before CX3CL1 (1 μg/ml) stimulation for 6 h. The expression of P-MLC was determined by immunoblotting. **(C)** The Western blotting results are presented as a histogram showing the band intensity values. **(D)** After transfection with P115-RhoGEF siRNA for 48 h, VMECs were treated with Y-27632 (10 μM) and/or Bosutinib (2 μM) for another 1h before CX3CL1 (1 μg/ml) stimulation for 6 h. The expression of ZO-1 was determined by immunoblotting. *P < 0.05 vs. the negative control. ^#^*P* < 0.05 vs. the corresponding CX3CL1. NS, no significance.

To further clarify the role of the Src/P115-RhoGEF/ROCK signaling pathway in CX3CL1-induced EC monolayer barrier disruption. We measured the flux changes in EB-albumin in a dual-chamber system after incubating VMECs with CX3CL1. We found that CX3CL1 increased the flux of EB-albumin from the upper chamber to the lower chamber and significantly induced VMEC barrier disruption. The inhibition of the Src/P115-RhoGEF/ROCK signaling pathway effectively inhibited CX3CL1-induced EC barrier dysfunction (Figure 12A). We also found that the inhibition of the Src/P115-RhoGEF/ROCK signaling pathway blocked CX3CL1-induced P-MLC expression and F-actin formation in VMECs (Figures 12B,C). Moreover, in this study, we found that the inhibition of the Src/P115-RhoGEF/ROCK signaling pathway reversed CX3CL1-induced ZO-1 disruption (Figure 12D). These data strongly showed that the Src/P115-RhoGEF/ROCK signaling activation plays an important role in CX3CL1-induced VMEC stress fiber formation, ZO-1 disruption and subsequent EC monolayer barrier disruption.

### Inhibiting Src/P115-RhoGEF/ROCK Signaling in VMECs Reverses CX3CL1-Induced Tumor Cell TEM

**FIGURE 13:**
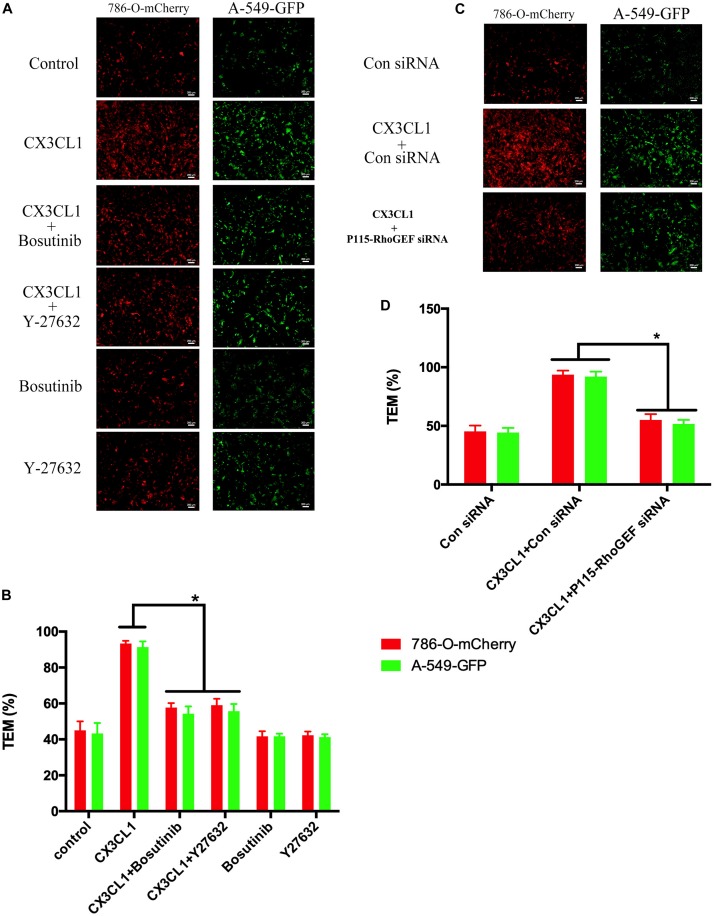
Effect of Src/P115-RhoGEF/ROCK signaling pathway on tumor cells TEM. **(A)** After pre-treating with Y-27632 (10 μM) or Bosutinib (2 μM) for 1 h, VMECs were treated with CX3CL1 (1 μg/ml) for 6h, and the TEM was determined using fluorescently labeled cancer cells (7860-O-mCherry and A-549-GFP) in the Transwell chamber. **(B)** The TEM rate of fluorescently labeled cancer cells was presented as a histogram. *P < 0.05 vs. the corresponding CX3CL1. **(C)** After transfection with P115-RhoGEF siRNA for 48 h, VMECs were treated with CX3CL1 (1 μg/ml) for 6 h, and the TEM was determined using fluorescently labeled cancer cells (7860-O-mCherry and A-549-GFP) in the Transwell chamber. **(D)** The TEM rate of fluorescently labeled cancer cells was presented as a histogram. *P < 0.05 vs. the corresponding control siRNA group.

The hyperpermeability of VMEC monolayers in the vertebral spongy bone promotes trans-endothelial migration (TEM) of circulating tumor cells that were arrested in the microvasculature in the spine. To determine the role of VMEC monolayers on tumor cell TEM in spines loaded with CX3CL1, we used fluorescently labeled lung cancer cells (A-549) and renal cancer cells (786-O) to assess changes in the TEM rate of cancer cells after regulating the activity of Src/P115-RhoGEF/ROCK signaling in VMECs. We found that only incubating VMECs with CX3CL1 increased the TEM of tumor cells but that pre-inhibition of Src/P115-RhoGEF/ROCK signaling in the VMECs significantly reversed CX3CL1-induced VMECs barrier hyperpermeability and the high rate of tumor cell TEM (Figures 13A,C). The TEM rate of tumor cells is represented as histograms (Figures 13B,D), which clearly showed that Src/P115-RhoGEF/ROCK signaling played an important role in VMEC barrier dysfunction and tumor cell TEM in the spine.

## Discussion

Bone metastasis arises via a complex succession of events, which involves the dissemination and growth of different cancer cells to distant bone. The escaping of circulating tumor cells from the micro-vasculature of primary sites and extravasating into distant sites are the most important steps of the metastatic cascade. Our previous study showed that the vertebral marrow sinusoids were full of CX3CL1, which plays an important role in the process of spine metastases derived from CX3CR1-expressing cancer cells (Liang et al., 2018; Liu et al., 2018). During the above process, CX3CL1 chemo-attracted tumor cells and functioned as an adhesion molecule to arrest circulating cancer cells in the vertebra (Fong et al., 1998; Yao et al., 2014). However, whether CX3CL1 further breaks the vertebral micro-vascular barrier and promotes tumor cell TEM remains unknown. In the present study, our results demonstrate that CX3CL1 induced VMEC barrier disruption in a dose- and time-dependent manner. Importantly, CX3CL1-induced stress fiber formation in VMECs via activation of the Src/p115-RhoGEF/ROCK signaling pathway, which plays an important role in VMEC monolayer hyper-permeability and tumor cell high TEM in the spine.

Chemokines serve critical roles in the progression of tumor metastasis. CX3CL1 is involved in the invasion and metastasis of various malignant tumor types and promotes tumor metastasis via chemotaxis and adhesion of circulating tumor cells (Tardáguila et al., 2013; Liu et al., 2016). Our previous study found that CX3CL1 was highly expressed in spinal osseous tissues compared with limb osseous tissues, and the patients with spinal metastases exhibited high levels of CX3CL1 (Liu et al., 2017). The above results suggested that CX3CL1 serves essential roles in tumor cell metastasis to the spine. However, the mechanisms by which CX3CL1 in vertebrae mediates tumor cell spine metastasis remain unclear. Some studies have reported that CX3CL1 promoted aortic endothelial dysfunction during heart failure (Schäfer et al., 2007; Hildemann et al., 2014). However, CX3CL1 involvement in the TEM of tumor cells by regulating vertebral micro-vascular endothelial barrier function has not yet been reported in the literature. In this study, we used TER technology to dynamically monitor the permeability changes in VMECs after incubating with different doses of CX3CL1. We found that CX3CL1 induced decreased TER in a dose- and time-dependent manner in VMECs. These data suggested that CX3CL1 effectively promoted VMEC monolayer barrier disruption and hyper-permeability.

Vascular barrier function depends on the status of centripetal tension leading to endothelial cell retraction (Michel and Curry, 1999). Centripetal tension is generated by actin/myosin cross-bridging and stress fiber formation (Bogatcheva and Verin, 2008). The actin filaments are approximately 7 nm in diameter and are composed of two strands of spiral fiber. These filaments are important structures of the protein fiber network in cells and act to maintain cell shape and cell barrier integrity (Xue et al., 2019). Actin monomers link with each other to form an actin chain. This actin polymer is filamentous actin (F-actin). The main functions of F-actin are the formation of stress fibers, cell contraction and cell barrier disruption (Gao et al., 2017). However, whether CX3CL1 induced vertebral vascular barrier disruption via the formation of F-actin in VMECs is unclear. Phosphorylation of MLC by MLC kinase (MLCK) promotes myosin filament assembly and F-actin formation (Pedersen and Hoffmann, 2002). In the present study, we found that CX3CL1 increased the expression of P-MLC in VMECs. We also found that CX3CL1 significantly induced a large amount of F-actin formation in VMECs via immunofluorescence methods. Furthermore, the ZO-1 which is belong to TJs acts as an intracellular scaffolding protein in ECs and regulates cell-cell tension and cytoskeletal organization (Matter and Balda, 2003). Our previous study has showed that ZO-1 plays an important role in the integrity of EC barrier (Yi et al., 2017). However, whether CX3CL1 induced vertebral vascular barrier disruption via the disruption of ZO-1 in VMECs is unclear. In the present study, we found that CX3CL1 decreased the expression of ZO-1 in VMECs. We also found that CX3CL1 significantly induced the disruption of ZO-1 in VMECs via immunofluorescence methods. These data suggested that CX3CL1-induced VMEC stress fibers formation and ZO-1 disruption play key roles in vertebral vascular barrier dysfunction.

Src signaling is implicated in the regulation of several pathways and plays an important role in cell migration via the regulation of the cytoskeleton (Hamaguchi et al., 1995). Moreover, Src signaling activation increases LPS-mediated microvascular permeability (Gong et al., 2008). Src activation is achieved by tyrosine 416 phosphorylation (Hunter, 1987). To explore the effects of Src signaling on CX3CL1-induced VMECs barrier disruption, the expression of total and phosphorylated Src was detected. In the present study, treatment with CX3CL1 increased the expression of P-Src (Tyr416) in a dose- and time-dependent manner in VMECs. This result suggested that the P-Src (Tyr416)-mediated activation of Src signaling might be implicated in CX3CL1-induced VMECs barrier disruption. Furthermore, inactivation of Src via special inhibitors effectively reversed CX3CL1-induced F-actin formation and ZO-1 disruption in VMECs, which indicated that Src signaling played an essential role in stress fiber formation, TJs disruption and subsequent VMECs barrier dysfunction.

It is well known that ROCK kinase plays an important role in the stress fiber and paracellular gap formation in ECs (Essler et al., 2000; Tasaka et al., 2005). Our previous study found that the activation of GEF-H1/ROCK signaling positively regulated LPS-induced ECs injury (Zhou et al., 2013; Yi et al., 2019, 2020). Interestingly, other scholars reported that P115-RhoGEF contributed to ROCK activation in vascular hypertension (Ying et al., 2004). In this study, we observed that CX3CL1 rapidly activated both P115-RhoGEF/ROCK and GEF-H1/ROCK signaling pathway in VMECs. A study by Scott et al. (2016) showed that SFKs regulated P115-RhoGEF in response to ECs tension on junctional adhesion molecule A. In light of this, we further used a specific Src inhibitor to inhibit the activity of Src and monitored changes to P115-GEF and GEF-H1. We found that the inhibition of Src activity significantly blocked the CX3CL1-induced activation of P115-RhoGEF/ROCK signaling but did not block CX3CL1-induced expression of GEF-H1. These data reveal that the Src/P115-RhoGEF/ROCK signaling pathway might be involved in CX3CL1-induced VMEC barrier disruption.

Thrombin promotes human monocytic cell F-actin cytoskeletal remodeling and migration via P115-RhoGEF (Gadepalli et al., 2013). Moreover, ROCK kinase quickly induced aberrant cytoskeletal organization in HeLa cells (Wu et al., 2016). Consistent with these findings, we observed that inhibition of P115-RhoGEF/ROCK signaling effectively blocked the CX3CL1-induced increase in P-MLC expression and stress fiber formation in VMECs. We also found that inhibition of P115-RhoGEF/ROCK signaling reversed the CX3CL1-induced decrease of ZO-1 expression and TJs disruption in VMECs. Furthermore, we found that the co-inhibition of Src and P115-RhoGEF and the co-inhibition of Src and ROCK did not promote augmented inhibiting effects on CX3CL1-induced F-actin formation and ZO-1 disruption. These data suggested that Src, as an upstream effector, is essential for P115-RhoGEF/ROCK signaling-mediated stress fiber formation and TJs disruption in VMECs after exposure to CX3CL1.

In this study, we further found that CX3CL1-induced ECs barrier disruption and extravasation of EB-albumin into the lower chamber were significantly decreased by inhibition of the Src/P115-RhoGEF/ROCK signaling pathway, which indicated that Src/P115-RhoGEF/ROCK signaling plays a critical role in regulating CX3CL1-induced VMEC barrier disruption and EC monolayer hyper-permeability. To further characterize whether the CX3CL1-induced activation of Src/P115-RhoGEF/ROCK signaling in VMECs plays an important role in the TEM of cancer cells, we monitored changes in the TEM rate of fluorescently labeled tumor cells across VMEC monolayers. We found that incubation VMECs with CX3CL1 significantly increased the migration rate of cancer cells and that inhibiting Src/P115-RhoGEF/ROCK signaling in VEMCs effectively blocked the process of tumor cell TEM. These data together with the results of previous studies (Furio et al., 2018; Ren et al., 2019) indicated that in addition to their roles in chemotaxis and adhesion, CX3CL1-mediated VMECs barrier disruption and subsequent vertebral micro-vascular hyper-permeability also play key roles in cancer cell TEM in the spine.

Our findings showed that Src/P115-RhoGEF/ROCK signaling exerts its vascular endothelial barrier disruptive effect by regulating the formation of F-actin and disruption of ZO-1 in CX3CL1-induced VMECs. This is a previously unreported mechanism by which CX3CL1 contributes to the vertebral micro-vascular barrier dysfunction and promotes tumor cell TEM to the spine. Our results suggest that the beneficial effects of the inactivation of the Src/P115-RhoGEF/ROCK signaling pathway in VMECs may be considered as new therapies for the treatment of tumor cell TEM in the spine.

## Data Availability Statement

The datasets generated for this study are available on request to the corresponding author.

## Ethics Statement

The animal study was reviewed and approved by the Scientific Investigation Board of Shanghai Jiao Tong University School of Medicine, Shanghai, China (SYXK2018-0027).

## Author Contributions

LY and YL made substantial contributions to analysis and interpretation of data, drafting the article and revising it critically for important intellectual content, and final approval of the version to be published. QZ and HW made substantial contributions to conception and design of the work and acquisition of data, editing the manuscript, and final approval of the version to be published. JD made substantial contributions to conception and design of the work and analysis and interpretation of data, drafting and revising the manuscript critically for important intellectual content, and final approval of the version to be published.

## Conflict of Interest

The authors declare that the research was conducted in the absence of any commercial or financial relationships that could be construed as a potential conflict of interest.
